# Cardiovascular and renal comorbidities among saudi patients with type 2 diabetes: A cross-sectional observational study

**DOI:** 10.1371/journal.pone.0324233

**Published:** 2025-05-27

**Authors:** Syed Irfan Karim, Kamran Sattar, Tauseef Ahmad, Mustafa Kamal Memon, Abdulfattah S. Alqahtani, Abdulrahman M. Alsubiheen, Fahad Abdulaziz Alrashed

**Affiliations:** 1 Department of Family and Community Medicine, College of Medicine, King Saud University, Riyadh, Saudi Arabia; 2 Department of Medical Education, College of Medicine, King Saud University, Riyadh, Saudi Arabia; 3 Department of Health Rehabilitation Sciences, College of Applied Medical Sciences, King Saud University, Riyadh, Saudi Arabia; 4 Department of Medical Education, College of Medicine, King Saud University, Riyadh, Saudi Arabia; District Head Quarter (DHQ) Hospital Charsadda / University of Peshawar, PAKISTAN

## Abstract

**Objective:**

This study aims to investigate the prevalence and patterns of cardiovascular and renal complications among patients with T2DM in the Saudi population and elucidate the extent of these comorbidities and their potential risk factors.

**Methods:**

A cross-sectional observational study was conducted across three research locations in Riyadh, Saudi Arabia. The study incorporated the first 248 T2DM patients who met the criteria and provided their consent. The sites for this research comprised one secondary care public hospital, one public primary care clinic, and one private medical facility. Efforts were made to evenly distribute patients across the six locations, spanning three distinct sectors. Should any location fall short of its patient target, other sites would step in to balance the deficit. Patient data was gathered during their enrollment visit as well as the patient’s medical records. These encompassed variables such as age, gender, race, smoking status, residential location, duration of T2DM, most recent HbA1c, blood pressure, lipid levels, kidney function, and most recent weight/body mass index (BMI).

**Results:**

Those with diabetes for five years or longer were more likely to have CKD (2.1 times higher), CAD (3.2 times higher), cerebrovascular disease (4.3 times higher), and hypertension (6.2 times higher). Most participants knew diabetes was a common health problem, and those with diabetic relatives were at a higher risk. In the present study, patients with uncontrolled HbA1C diabetes demonstrated a notably increased prevalence of various comorbidities CKD (OR=3.9, p < 0.0001), CAD (OR=2.3, p = 0.007), CHF (OR=3.1, p = 0.0001), cerebrovascular disease (OR=2.4, p = 0.0004), CVD (OR=4.2, p=<0.0001) and hypertension (OR=3.5, p = 0.0001) compared to those without uncontrolled HbA1C diabetes. However, CVD and hypertension shows a stronger association with diabetes The analysis demonstrated that diabetes was highly correlated to neuropathy (t = 2.204, p = 0.002), coronary artery disease (t = 1.53, p = 0.03), congestive heart failure (CHF) (t = 1.34, p = 0.05), cerebrovascular Disease (t = 2.65, p = 0.009), and hypertension (t = 5.05, p = 0.000).

**Conclusion:**

We concluded that patients who had diabetes for five years or more had considerably greater risks of developing comorbidities such as chronic kidney disease, coronary artery disease, cerebrovascular disease, and hypertension. Among others hypertension being a major comorbidity that significantly influences the progression or presence of diabetes. This highlights the necessity of beginning treatment as early as possible and maintaining glycemic control to reduce the risk of diabetes-related problems in the long run. One limitation of this study is its cross-sectional design, which only captures data at a single point in time, preventing the establishment of causal relationships between variables.

## Introduction

Type 2 diabetes mellitus (T2DM) is a chronic metabolic disorder characterized by hyperglycemia resulting from insulin resistance and insufficient insulin secretion. Its prevalence has been steadily rising worldwide, making it a significant global health concern. According to a study, the global number of people aged 18 and older living with type 2 diabetes (T2DM) has reached 422 million, which represents an approximate prevalence of 8.5%. Remarkably, the highest rates of T2DM are found in middle- and low-income nations, where the incidence is steadily increasing [1–4]. The burden of T2DM is further compounded by its association with various cardiovascular and renal complications, which significantly contribute to morbidity and mortality among affected individuals[5,6]. Individuals with diabetes face a 2–4 times greater risk of developing heart disease or experiencing a stroke compared to those without diabetes. Additionally, at least 68% of diabetic patients aged 65 and older are projected to die from heart disease, while 16% are anticipated to succumb to a stroke [7]. Saudi Arabia, like many other countries, has witnessed an alarming increase in the prevalence of T2DM, warranting a comprehensive understanding of the concomitant cardiovascular and renal comorbidities. Approximately 7 million people in Saudi Arabia are diagnosed with diabetes, with an additional 3 million classified as pre-diabetic[8]. The national healthcare cost attributed to diabetes in Saudi Arabia is projected to surpass $0.87 billion[9]. According to the IDF Diabetes Atlas (Saudi Arabia Diabetes Report 2000–2045), the total number of diabetes-related deaths in Saudi Arabia by 2021 is estimated to reach 32,054 [10]. Saudi Arabia is experiencing an escalating prevalence of T2DM, driven by multiple factors including urbanization, sedentary lifestyles, and changing dietary habits [11,12]. Recent epidemiological studies have highlighted the growing burden of T2DM in the Saudi population, with a prevalence of approximately 17.6% in adults aged 30–70 years [13]. Patients with T2DM have a substantially increased risk of developing various CVDs, including coronary artery disease, stroke, and peripheral vascular disease [14,15]. The underlying mechanisms linking T2DM to CVD are complex and involve factors such as insulin resistance, inflammation, dyslipidemia, and endothelial dysfunction. These factors collectively contribute to accelerated atherosclerosis and an elevated risk of adverse cardiovascular events [16–18].

Renal complications, particularly diabetic nephropathy, are common in individuals with T2DM. Diabetic nephropathy is a leading cause of end-stage renal disease (ESRD), necessitating dialysis or renal transplantation [19,20]. The prevalence of diabetic nephropathy is notably higher in T2DM patients compared to those with type 1 diabetes, and it is closely intertwined with the duration and severity of diabetes [21]. The intricate interplay between hyperglycemia, inflammation, and hemodynamic alterations contributes to the development and progression of diabetic nephropathy [22,23]. While substantial research has been conducted globally on the prevalence of cardiovascular and renal comorbidities in patients with Type 2 Diabetes Mellitus (T2DM), there remains a notable gap in the understanding of this phenomenon within the context of Saudi Arabia. The available literature predominantly focuses on Western populations, and limited attention has been directed towards investigating the specific prevalence and patterns of cardiovascular and renal complications among Saudi Arabian individuals with T2DM.

This research gap is of significant importance due to the unique demographic, cultural, and lifestyle factors that could influence the epidemiology of T2DM-related comorbidities in Saudi Arabia. Additionally, while some studies have reported the prevalence of T2DM and its complications in Saudi Arabia, there is a scarcity of research that delves deeper into the risk factors, disease trajectories, and potential interventions to mitigate the burden of cardiovascular and renal comorbidities among T2DM patients. Understanding the specific risk factors that are more prominent within the Saudi Arabian population, such as consanguinity, dietary habits, and genetic markers, could provide crucial insights for tailored preventive strategies.

To bridge this research gap, a comprehensive study that investigates the prevalence, risk factors, and patterns of cardiovascular and renal comorbidities in patients with T2DM in Saudi Arabia is warranted. This study aims to investigate the prevalence and patterns of cardiovascular and renal complications among patients with T2DM in the Saudi population and elucidate the extent of these comorbidities and their potential risk factors.

## Methods

### Study design

Between 17 January 2024 and 30 June 2024, a cross-sectional observational original study was conducted across three research locations in Riyadh, Saudi Arabia. The study incorporated the first 248 T2DM patients who met the criteria and provided their consent. The sites for this research comprised one secondary care public hospital, one public primary care clinic, and one private medical facility. Efforts were made to evenly distribute patients across the six locations, spanning three distinct sectors. Should any location fall short of its patient target, other sites would step in to balance the deficit. Patient data was gathered during their enrollment visit as well as the patient’s medical records. A team of five highly skilled researchers conducted comprehensive health assessments at the research sites and administered structured questionnaire-based interviews (Appendix 1), adhering to a standardized data collection protocol. The collected data encompassed measurements of vital health metrics such as blood pressure, weight, and height. Hospital records supplemented the dataset with crucial information, including participant age, disease duration, comorbidities, and cholesterol levels. This stringent adherence to protocol was essential to ensure data validity and reliability. All the researchers who collected data were final-year medical students enrolled in MBBS programs, with uniform and rigorous training to perform these assessments. In cases where data omissions or errors were identified, additional interviews or examinations were conducted to rectify any discrepancies.

### Study population

A total of 248 residents from Saudi Arabia, all diagnosed with T2DM, were enrolled during the specified timeframe at the study sites. The investigator or a qualified designee meticulously assessed each patient’s eligibility, reviewing both inclusion and exclusion criteria. This was done to ensure their suitability for participation. Patients were identified as having the target disease based on documented diagnoses. The study inclusion criteria included patients who were required to have received a prior T2DM diagnosis from a physician before enrolling. Participants needed to be eighteen years old at their enrollment visit. Patients must have visited the study site at least once between January 17, 2024, and June 30, 2024. Additionally, individuals were eligible if they had a clinical record at the healthcare center spanning at least one year before their enrollment. Exclusion criteria for the study included: individuals with gestational diabetes mellitus. Patients with other forms of secondary diabetes were also not eligible. Those unable to provide informed consent or effectively communicate, including mental illness patients, were excluded from participation. After reviewing all the collected data, the research team checks for any missing data. If missing data is identified, those cases are excluded from the final dataset to ensure the integrity of the analysis.

### Sample Size

Our estimation anticipated the recruitment of approximately 250–300 patients within the study sites during the designated index period.

Prevalence cardiovascular and renal comorbidities among patients with type 2 diabetes mellitus

in literature review:

- A previous study by E Al-ozairi, et al 2021[24];” *Co-morbidities in patients with type2 diabetes in the gulf*” used 32%

- Another study by A.H Al-jedai, et al.2022[25]; *“Renal complications in people with type 2 diabetes mellitus in the Kingdom of Saudi Arabia*” used 33.4%

**Based on the previous study our sample size:**

We used a maximum prevalence of 33.4% or (0.334)

d= Absolute error 5%

Sample size formula = **Z²x(p(1-p))/d²**

Sample size= 1.96^2^ * (0.334(1-0.334))/0.05² = 291

Sample size if the prevalence is 33.4% according to a previous published study = 292

### Ethics approval and consent to participate

This study was approved by the Institutional Review Board (IRB) at King Saud University (KSU) in Riyadh, Saudi Arabia (#E-23–8179). Before participating, all participants signed a consent form. All participants provided informed consent prior to data collection. Participation was voluntary. The methods used were in accordance with all relevant guidelines and regulations.

### Statistical analysis

The primary objective of the study was to assemble a diverse cohort of T2DM patients from the selected sites in Saudi Arabia. Baseline demographic data and clinical characteristics were described using descriptive statistics. These encompassed variables such as age, gender, race, smoking status, residential location, duration of T2DM, most recent HbA1c, blood pressure, lipid levels, kidney function, and most recent weight/body mass index (BMI). Data conforming to a normal distribution were presented using means and standard deviations, while those displaying non-normal or skewed distributions were presented with medians and interquartile ranges, following a non-parametric distribution approach. To explore the association between two comorbidities, correlation coefficients were employed as a measure of strength. A two-sided significance level with a type 1 error rate of 5% was applied throughout the analyses. All statistical analyses were performed using the Statistical Package for Social Sciences version 24 (SPSS-24).

## Results

We invited 436 diabetic patients to participate in the current study over a period of eight months. However, only 248 patients agreed to give written consent to participate in the current study. There were 233 Saudi participants (94%) and only 15 non-Saudi participants (6%) among them. Male participants were 120(48.4%) and female participants 128 (51.6%) in the current study Most of the patients (78.2%) had type 2 diabetes. There was a median (IQR) weight of 70 kilograms in the age group of 18–50 years, 85 kilograms in the age group of 51–65 years, and 77.5 kilograms in the age group of 66 or more years. Additionally, as compared to other systolic blood pressure age groups, the systolic blood pressure was higher in the age group of 66 or more years (IQR = 144 mmHg). Furthermore, the median (IQR) diastolic blood pressure was higher in the younger age group (IQR = 80 mmHg) than in other older age groups. The median HbA1c was higher (IQR = 8.7%) in the age 18–50 years of the population than other age groups. The median (IQR) total cholesterol and low-density lipoprotein were higher at 5.35 (2.99–6.50) mg/dl, and 2.88 (1.13–4.25) mg/dl in the age group of 66 and older, respectively. In a total of 36 diabetes patients, the median serum creatinine level was higher 56(5–178) mg/dl in the elderly group. In the present study, the majority of patients had diabetes with a comorbidity, 21% of patients had no comorbidity, and 26.6% of diabetic patients had a comorbidity (the highest rate being among those over the age of 66 years or more). Similarly, 26.6% of patients had two comorbid conditions, while 9.7% of patients had three comorbid conditions (Table 1).

**Table 1:** Description of clinical characteristics of the study population, by age group.

				18–50		51–65		66 or more	
Items	Categories	Not tested	n(%)	N (%), or Median (IQR)		N (%), or Median (IQR)		N (%), or Median (IQR)	
Nationality	Non-Saudi		15(6.0)	6 (5.3)		9(9.1)		0(0.0)	
	Saudi		233(94.0)	107(94.7)		90(90.9)		36(100)	
Gender	Male		120(48.4)	69(61.1)		42(42.4)		9(25.0)	
	Female		128(51.6)	44(38.9)		57(57.6)		27(75.0)	
Diabetes type	Type 1		54(21.8)	54(47.8)		0(0.0)		0(0.0)	
	Type 2		194(78.2)	59(52.2)		99(100.0)		36(100.0)	
Weight	KG		248(100)	113	70(52-138)	99	85(60-131)	36	77.5(58-103)
Height	CM	9	239(94.6)	104	163(145-179)	99	160(144-182)	36	156(150-168)
Systolic blood pressure	mmHg		248(100)	113	129(98-159)	99	138(109-177)	36	144(124-160)
Diastolic blood pressure	mmHg		248(100)	113	80(50-95)	99	74(60-90)	36	75.5(58-92)
HbAIC	%	3	245(98.8)	110	8.7(5.6-12.2)	99	7.8(5.3-14.0)	36	7.9(6.3-12.3)
Total cholesterol	mg/dl	2	246(99.2)	111	4.5(2.68-6.66)	99	5.05(2.50-7.82)	36	5.35(2.99-6.50)
LDL	mg/dl	2	246(99.2)	111	2.62(1-5.04)	99	2.78(0.70-4.90)	36	2.88(1.13-4.25)
HDL	mg/dl	2	246(99.2)	111	1.25(0.54-2.20)	99	1.39(0.59-1.89)	36	1.17(0.79-1.91)
Triglycerides	mg/dl	5	243(98.0)	111	1.13(0.46-3.80)	96	1.67(0.58-4.84)	36	1.16(0.77-2.15)
Serum creatinine	mg/dl	5	243(98.0)	108	66(35-126)	99	66(1-153)	36	56(35-178)
Urinary albumin/creatinine ratio	mg/dl	17	231(93.1)	102	10(1-171)	96	11.50(1-389)	33	36(5-787)
Comorbidities									
0 Comorbidity		1	52(21.0)	24(21.2)		22(22.2)		6(16.7)	
1 Comorbidity			66(26.6)	25(22.1)		24(22.1)		17(47.2)	
2 Comorbidity			66(26.6)	33(29.2)		27(27.3)		6(16.7)	
3 comorbidity			24(9.7)	11(9.7)		10(10.1)		3(8.3)	
4 Comorbidity			9(3.6)	4(3.5)		5(5.1)		0(0.0)	
5 Comorbidity			15(6.0)	7(6.2)		6(6.1)		2(5.6)	
6 comorbidity			15(8.0)	8(7.1)		5(5.1)		2(5.6)	

The median weight of male patients was 80.5 (55–138) KG, and the median weight of female patients was 77 (52–118) KG. There was a higher median (IQR) systolic blood pressure in females than in males, 135 (98–164) mmHg on average. The diastolic blood pressure of female patients in the current study was also high at 78 mmHg, compared with male patients. HbA1c levels were higher among male diabetes patients in the present study by 8.7% (5.3–14.0) than among female patients. Additionally, females had significantly higher levels of total cholesterol and LDL compared to males (median = 4.9 mg/dl; median = 2.7 mg/dl). Male diabetic patients had higher levels of triglycerides at 1.4 mg/dl than female diabetic patients. In the current study, female diabetic patients had more comorbidities (30.5% of females had one comorbidity; 29.7% of females had two comorbidities) than male diabetic patients (Table 2).

**Table 2:** Description of clinical characteristics of the study population, by gender.

				Male		Female	
Items	Categories	Not tested	n(%)	N (%), or Median (IQR)		N (%), or Median (IQR)	
Nationality	Non-Saudi		15(6.0)	15(12.5)		0(0.0)	
	Saudi		233(94.0)	105(87.5)		128(100.0)	
Age	18-50			69(57.5)		44(34.4)	
	51-66			42(35.0)		57(44.5)	
	66 or more			9(7.5)		27(21.1)	
Diabetes type	Type 1		54(21.8)	30(25.0)		24(18.8)	
	Type 2		194(78.2)	90(75.0)		104(81.3)	
Weight	KG		248(100)	120	80.5(55-138)	128	77(52-118)
Height	CM	9	239(94.6)	111	170(145-182)	128	156(144-171)
Systolic blood pressure	mmHg		248(100)	120	130.5(100-177)	128	135(98-164)
Diastolic blood pressure	mmHg		248(100)	120	74.5(63-95)	128	78(50-94)
HbAIC	%	3	245(98.8)	117	8.7(5.3-14.0)	128	7.9(5.9-14.0)
Total cholesterol	mg/dl	2	246(99.2)	120	4.7(2.5-7.8)	126	4.9(2.9-7.1)
LDL	mg/dl	2	246(99.2)	120	2.5(0.70-5.04)	126	2.7(1.26-4.90)
HDL	mg/dl	2	246(99.2)	120	1.17(0.54-2.0)	126	1.4(0.89-2.20)
Triglycerides	mg/dl	5	243(98.0)	120	1.4(0.46-4.84)	123	1.14(0.50-2.38)
Serum creatinine	mg/dl	5	243(98.0)	117	79(45-153)	126	55(1-178)
Urinary albumin/creatinine ratio	mg/dl	17	231(93.1)	108	10(1-787)	123	15(2-389)
Comorbidities							
0 Comorbidity		1	52(21.0)	29(24.2)		23(18.0)	
1 Comorbidity			66(26.6)	27(22.5)		39(30.5)	
2 Comorbidity			66(26.6)	28(23.3)		38(29.7)	
3 comorbidity			24(9.7)	14(11.7)		10(7.8)	
4 Comorbidity			9(3.6)	7(5.8)		2(1.6)	
5 Comorbidity			15(6.0)	7(5.8)		8(6.3)	
6 comorbidity			15(8.0)	7(5.8)		8(6.3)	

### Clinical Outcomes

Those with diabetes for five years or longer were more likely to have CKD (2.1 times higher), CAD (3.2 times higher), cerebrovascular disease (4.3 times higher), and hypertension (6.2 times higher). Most participants knew diabetes was a common health problem, and those with diabetic relatives were at a higher risk. According to the current study, CHF is 3.1% more common, CKD is 2.4 times more common, Cerebrovascular disease is 3.5 times more common, CVD is 2.1 times more common, and hypertension is 3.82 times more common among diabetic patients with a family history of diabetes. Diabetic and uremic patients had a significantly higher prevalence of CAD (2.17 times higher), CHF (3.51 times higher), and cerebrovascular disease (3.1 times higher). In the present study, patients with uncontrolled HbA1C diabetes demonstrated a notably increased prevalence of various comorbidities CKD (OR=3.9, p < 0.0001), CAD (OR=2.3, p = 0.007), CHF (OR=3.1, p = 0.0001), cerebrovascular disease (OR=2.4, p = 0.0004), CVD (OR=4.2, p=<0.0001) and hypertension (OR=3.5, p = 0.0001) compared to those without uncontrolled HbA1C diabetes. (Table 3)

**Table 3:** Association of diabetes risk level and comorbidities patients.

	CKD		CAD		CHF		PAD		Cerebrovascular disease		CVD		hypertension	
Factor	95%CI	p = value	95%CI	p = value	95%CI	p = value	95%CI	p = value	95%CI	P = value	95%CI	p = value	95%CI	p = value
**Duration (more than 5 year)**	2.1(1.7-2.6)	0.008	3.2(1.9- 5.1)	<0.0001	2.2(1.8- 2.7)	0.0005	1.6(0.73- 3.5)	0.09	4.3(2.3- 9.1)	<0.0001	1.3(0.6- 2.8)	0.01	6.2(03.1- 10.7)	<0.00001
**Family History**	2.4(1.9- 4.8)	0.001	1.9(0.72- 2.8)	0.03	3.1(1.9- 5.2)	0.00001	1.4(0.72- 2.8)	0.3	3.5(2.6- 4.8)	<0.0001	2.1(1.3- 3.3)	0.08	3.8(2.1- 8.5)	<0.00001
**Recurrent infections**	0.93(0.1- 1.4)	0.59	1.3(0.48- 2.3)	0.14	2.4(1.03-4.9)	0.005	1.13(0.9-1.4)	0.18	1.4(0.8- 2.6)	0.007	1.2(0.8- 1.7)	0.31	2.4(1.7- 3.6)	0.001
**Urea**	1.2(0.82- 2.0)	0.26	2.17(1.37- 3.4)	0.008	3.51(2.1- 4.9)	<0.0001	1.9(1.32- 2.7)	0.009	3.1(1.9- 4.6)	<0.0003	2.2(1.6- 3.6)	0.005	1.7(0.92- 2.7)	0.04
**HbA1C**	3.9(2.6- 5.6)	<0.0001	2.3(1.4- 3.1)	0.007	3.1(2.1-4.6)	0.0001	1.4(0.81- 2.2)	0.02	2.4(1.8- 3.2)	0.0004	4.2(3.1- 6.5)	<0.00001	3.5(2.2- 4.5)	0.0001
**Cholesterol**	1.1(0.6- 1.5)	0.48	3.8(2.6- 5.1)	<0.0001	2.38(1.7- 3.2)	<0.0001	1.57(1- 2.4)	0.06	4.1(3.2- 5.8)	<0.0001	4.3(3.3- 6)	<0.0001	5.1(3.9- 7.1)	<0.00001
**LDL**	1.4(0.8- 2.3)	0.14	2.2(1.3- 2.9)	0.02	2.8(1.9- 3.6)	0.002	1.8(1.1- 2.8)	0.05	2.9(2.1- 4.3)	0.0001	3.4(2.3- 4.6)	<0.0001	2.4(1.7- 3.2)	0.0031
**HDL**	0.7(0.48- 1.17)	0.21	0.8(0.5- 1.28)	0.28	0.67(0.4- 0.9)	0.003	1.1(0.71- 1.6)	0.66	1.2(0.9- 1.6)	0.11	1.3(0.9- 1.6)	0.06	1.4(0.8- 2.1)	0.04

The prevalence of diabetes with specific comorbidities was recorded and it was found that neuropathy showed a significant association with T2DM, with a χ² value of 12 (P = 0.000), indicating a strong relationship between T2DM and neuropathy. CVD also demonstrated a significant association with T2DM, with a χ² value of 7.39 (P = 0.002), suggesting T2DM patients are more likely to have CVD. CHF had a significant association, with a χ² value of 3.51 (P = 0.04), indicating that T2DM patients are at increased risk for CHF. In this current results also reported that cerebrovascular Disease showed a significant association, with a χ² value of 8.42 (P = 0.002), highlighting the higher prevalence of cerebrovascular issues among T2DM patients. Furthermore, hypertension had a very strong association with T2DM (χ² = 36.03, P = 0.000), with 57.2% of T2DM patients experiencing hypertension (Table 4). Diabetes patients had less effect (4.6%) on CAD as found in the current study. Diabetes showed an association with CHF (6.2%) which was significantly higher (p = 0.04). In our study, because there were intercorrelations between comorbidities, only the overall diabetes comorbidities levels domain was the only the independent variable integrated with the multiple linear regression model (Table 5). Analysis demonstrated that diabetes was highly correlated to neuropathy (t = 2.204, p = 0.002), CAD (t = 1.53, p = 0.03), CHF (t = 1.34, p = 0.05), CVD (t = 2.65, p = 0.009), and hypertension (t = 5.05, p = 0.000) (Table 5).

**Table 4:** Association of type of type 2DM with comorbidities.

Diabetes Comorbidities	Type of Comorbidities	Type 2 DM, not comorbidities	Type 2 DM	χ2 (P-value)
Comorbidities	Retinopathy	170(87.6)	24(12.4)	2.02(0.11)
	Neuropathy	155(79.9)	39(20.1)	12(0.000)
	CKD	176(90.7)	18(9.3)	0.16(0.42)
	CVD	170(87.6)	24(12.4)	7.39(0.002)
	CAD	185(95.4)	9(4.6)	2.59(0.10)
	CHF	182(93.2)	12(6.2)	3.51(0.04)
	PAD	173(89.2)	21(10.8)	1.34(0.18)
	Cerebra Vascular disease	149(76.8)	45(23.2)	8.42(0.002)
	Hypertension	83(42.8)	111(57.2)	36.03(0.000)
	Others*	71(36.6)	123(63.4)	107.6(0.000)

**Table 5:** Multiple-linear regression analysis of factors of comorbidities association with diabetes.

	Influencing factors of diabetes				
	Unstandardized coefficients		Standardized Coefficients		
Comorbidities variables	B	SE	Beta	*t-* value	*P*-value
Retinopathy	−0.174	0.093	−0.13	−1.87	0.062
Neuropathy	0.204	0.083	0.18	2.45	0.002
chronic kidney disease (CKD)	−0.414	0.102	−0.29	−4.06	0.000
cardio vascular disease (CVD)	−0.057	0.103	−0.041	−0.554	0.58
Coronary artery disease (CAD)	0.245	0.16	0.111	1.53	0.03
congestive heart failure (CHF)	0.177	0.13	0.092	1.34	0.05
Peripheral arterial disease(PAD)	0.009	0.087	0.007	0.105	0.91
Cerebrovascular Disease	0.212	0.08	0.203	2.65	0.009
Hypertension	0.299	0.059	0.362	5.055	0.00
Others*	0.009	0.054	0.011	0.17	0.865

## Discussion

This study explicitly has reflected the local prevalence of diabetes and its management practices, which may differ from those in other countries due to cultural, genetic, or healthcare system-related factors [8,11,26]. The gender distribution among participants was approximately balanced, with 48.4% being male and 51.6% female. This gender parity is consistent with the global trend of diabetes affecting both sexes relatively equally [27]. However, it is noteworthy that diabetes can impact men and women differently, with variations in risk factors and disease management [28–30]. Men and women exhibit different hormonal profiles, which can influence the development and progression of diabetes. Estrogen has protective effects against insulin resistance in women, especially pre-menopause, while men tend to have a higher risk of developing abdominal obesity, which is a strong diabetes risk factor [31,32]. Lifestyle factors such as diet and physical activity also play distinct roles in each gender, further influencing diabetes outcomes [33].

The substantial majority of patients (78.2%) having type 2 diabetes aligns with global epidemiological data indicating that type 2 diabetes is the most prevalent form of diabetes among adults [34]. The prevalence of type 2 diabetes often relates to lifestyle factors, such as diet and physical activity, as well as genetic predisposition [35]. The analysis of weight across different age groups revealed an interesting trend. Weight tended to increase with age, as evidenced by the higher median weight in the 51–65-year and 66 or more years age groups. This observation is in line with previous research highlighting age-related changes in body composition and metabolism, which can contribute to weight gain [36,37]. The age-related variation in weight underscores the importance of considering age as a potential covariate in future analyses investigating the relationships between weight, diabetes control, and related complications.

Systolic blood pressure was notably higher in the age group of 66 or more years, while diastolic blood pressure was higher in the younger age group. These findings emphasize the age-related differences in blood pressure, which is consistent with the established understanding that systolic blood pressure tends to increase with age, while diastolic blood pressure may exhibit a different pattern [38]. Elevated systolic blood pressure is a significant risk factor for cardiovascular events and complications in diabetic patients [39], and, therefore, the age-related variations in blood pressure warrant further investigation to assess their impact on health outcomes. The findings of this study provide valuable insights into the clinical characteristics and comorbidity patterns among diabetic patients within different age groups. Notably, HbA1c levels were highest in the 18–50-year age group, indicating poorer glycemic control in younger patients. This observation suggests a need for targeted interventions and education programs aimed at improving diabetes management in this age category. Total cholesterol and low-density lipoprotein (LDL) levels were elevated in individuals aged 66 and older. This age-related increase in lipid levels may contribute to a higher risk of cardiovascular complications among older diabetic patients [15,40,41].

Triglyceride levels were notably elevated in the 51–65-year age group, underscoring the importance of monitoring and managing lipid profiles across different age categories. Elevated triglycerides are associated with an increased risk of cardiovascular disease, particularly in middle-aged individuals [26,42]. Serum creatinine levels were higher in the elderly group, indicating a potential age-related decline in kidney function. Diabetic patients are at heightened risk of kidney complications, and these findings reinforce the need for vigilant monitoring and early intervention, especially in older individuals [43]. Comorbidity patterns revealed that a majority of diabetic patients had at least one comorbid condition, with the highest comorbidity rate observed in individuals aged 66 years and older. This highlights the complex healthcare needs of older diabetic patients and the importance of a comprehensive and multidisciplinary approach to their care [34,44]. Further, Iglay et al. found that CKD, CVD, and CHF were the most prevalent comorbidities among US patients with T2DM, with a prevalence of 24.1%, 21.6%, and 7.4%, respectively. The prevalence of multiple comorbid conditions, with some patients having three or more, underscores the challenges in managing diabetes in the presence of additional health issues. Such patients may require tailored treatment plans and closer medical supervision to mitigate the cumulative risks associated with multiple comorbidities [15,45,46].

This study highlights notable gender-related differences in the clinical characteristics of diabetic patients. Females had a slightly higher median weight compared to males, which could be influenced by various factors, including hormonal differences [47]. Remarkably, females exhibited higher systolic and diastolic blood pressure levels, indicating a potential increased risk of hypertension-related complications among female diabetic patients [15,48,49]. Contrastingly, males presented higher HbA1c levels, suggesting that glycemic control might be more challenging for them. This gender discrepancy in HbA1c aligns with previous research indicating variations in diabetes management between men and women [28]. Furthermore, females had significantly higher levels of total cholesterol and LDL, which may contribute to a higher risk of cardiovascular complications [40]. In contrast, males had elevated triglyceride levels, which could also impact cardiovascular health [15,42]. The findings indicate that female diabetic patients had a higher burden of comorbidities, suggesting a need for tailored care plans. This gender-specific variation in comorbidity rates underscores the importance of addressing not only diabetes but also its associated conditions in clinical management [45].

This study found patients with diabetes for five years or longer exhibited significantly higher risks of comorbidities, including CKD, CAD, cerebrovascular disease, and hypertension. This emphasizes the importance of early intervention and sustained glycemic control to mitigate the long-term complications of diabetes [50–52]. Patients with a family history of diabetes were at an increased risk of developing comorbidities, including CHF, CKD, cerebrovascular disease, CVD, and hypertension. Genetic predisposition and shared lifestyle factors within families may contribute to these associations [52,53]. Diabetic patients with uremia had notably higher prevalence rates of CAD, CHF, and cerebrovascular disease. In the current study we found lower prevalence of CAD in Saudi Arabia, compared to global data, could be attributed to regional differences in genetic predisposition, lifestyle factors, and healthcare access. Studies have shown that dietary habits, such as higher consumption of traditional foods and lower rates of smoking, may contribute to this difference [54]. Additionally, the younger population in Saudi Arabia and differences in diagnostic practices may also influence the observed prevalence [31,55].

This underscores the intricate relationship between kidney function and cardiovascular health in diabetes [56]. Patients with uncontrolled HbA1C diabetes demonstrated a significantly elevated prevalence of various comorbidities, emphasizing the critical role of glycemic control in reducing the risk of complications. This aligns with established evidence highlighting the importance of HbA1C management in diabetes care [13,57]. The study also examined the prevalence of specific comorbidities among diabetic patients. Neuropathy and hypertension were notably prevalent, highlighting the need for comprehensive screening and management strategies for these conditions. In addition, nephropathy was the most common combined complications (20.1%). The prevalence of suboptimal renal profiles was high, but only 5.92% of patients had nephropathy[58]. There is a strong correlation between hypertension and diabetes major comorbidity that significantly influences the progression or presence of diabetes.

Additionally, the relatively lower prevalence of coronary artery disease in this cohort warrants further investigation [59]. Multiple linear regression analysis revealed strong correlations between diabetes and neuropathy, coronary artery disease, congestive heart failure, cerebrovascular disease, and hypertension. This underscores the interconnectedness of these comorbidities and their shared risk factors in diabetes patients [35,57,59].

## Conclusion

Complications and individuals affected by T2DM are a focus of attention in the medical and healthcare communities. Its prevalence has been continuously rising all over the world, which makes it a big issue for the health of people all over the world. This study contributed to a more in-depth investigation and examination of the prevalence, risk factors, and patterns of cardiovascular and renal comorbidities connected to T2DM in Saudi patients. We concluded that patients who had diabetes for five years or more had considerably greater risks of developing comorbidities such as chronic kidney disease, coronary artery disease, cerebrovascular disease, and hypertension. This highlights the necessity of beginning treatment as early as possible and maintaining glycemic control in order to reduce the risk of diabetes-related problems in the long run. At every level of healthcare, both in the private and public sectors, more attention needs to be paid to the appropriate treatment of T2DM to reduce the risks that are associated with this widespread but preventable health condition, before it is too late and predisposes into renal and cardiovascular comorbidities.

### Limitation

One limitation of this study is its cross-sectional design, which only captures data at a single point in time, preventing the establishment of causal relationships between variables. The reliance on medical records and self-reported data may introduce recall bias or inaccuracies, especially concerning comorbidities and disease duration. Although efforts were made to ensure balanced patient distribution across sites, potential site-specific differences in healthcare access or diagnostic practices could influence findings. Furthermore, the exclusion of patients with mental illness or those unable to provide informed consent may limit the generalizability of the results to this subgroup.

## Supporting information

S1 Appendix 1 Questionnaire. (DOCX)

## Abbreviations

T2DM: Type 2 Diabetes Mellitus
CKD: Chronic Kidney Disease
CAD: Coronary Artery Disease
CHF: Congestive Heart Failure
CVD: Cardiovascular Disease
ESRD: End-Stage Renal Disease
MBBS: Bachelor of Medicine, Bachelor of Surgery
BMI: Body Mass Index
LDL: Low-Density Lipoprotein
KSU: King Saud University
IRB: Institutional review board
SPSS: Statistical package for the social sciences
