# Supplementary material for: Cardiovascular and renal comorbidities among saudi patients with type 2 diabetes: A cross-sectional observational study
Source: PLoS One. 2025 May 27;20(5):e0324233. doi: 10.1371/journal.pone.0324233 (PMC12112348; doi:10.1371/journal.pone.0324233)
Supplement: S1 Appendix 1 — (DOCX) [file pone.0324233.s001.docx]

**QUESTIONAIRRE BASED INTERVIEWS**

Hospital _______________________________________

Nationality _______________________________________

Gender _______________________________________

Age or DOB _______________________________________

Year of Diagnosis (of Diabetes) OR Duration in Years ______

Diabetes Type _______________________________________

Weight (Kg) _______________________________________

Height (cm) _______________________________________

Blood Pressure (Systolic) __________________________

Blood Pressure (Diastolic) __________________________

HbA1c (% or mmol/mol) __________________________

Total cholesterol __________________________

LDL __________________________

HDL __________________________

Triglyceride __________________________

Serum creatinine __________________________

eGFR __________________________

Urine Albuminuria __________________________

Had Bariatric surgery/sleeve gastrectomy? Yes / No

Comorbidities Type [Retinopathy] Yes / No

Comorbidities Type [Neuropathy] Yes / No

Comorbidities Type [CKD (chronic kidney disease)] Yes / No

Comorbidities Type [CVD (cardio vascular disease)] Yes / No

Comorbidities Type [CAD (coronary artery disease)] Yes / No

Comorbidities Type [CHF (congestive heart failure)] Yes / No

Comorbidities Type [PAD (peripheral arterial disease)] Yes / No

Comorbidities Type [Cerebrovascular Disease] Yes / No

Comorbidities Type [Hypertension] Yes / No

Other (if any comorbidities) ___________________________

CKD stage (if any) ___________________________

Recommended Medicine [Metformin] Yes / No

Recommended Medicine [Sulfonyl Urea] Yes / No

Recommended Medicine [Alpha glucosidase inhibitors] Yes / No

Recommended Medicine [DPP4 inhibitor (Januvia, Galvus)] Yes / No

Recommended Medicine [GLP-1RA (Victoza, Trulicity)] Yes / No

Recommended Medicine [SGL2 inhibitors (Forxiga, Jardiance)] Yes / No

Recommended Medicine [Meglitinides] Yes / No

Recommended Medicine [Thiazolidinediones] Yes / No

Recommended Medicine [Insulin] Yes / No

Recommended Medicine [Others] Yes / No

Note _____________________________________________________________

_____________________________________________________________

_____________________________________________________________
